# Effects of behavioral observability and social proof on the coupled epidemic-awareness dynamics in multiplex networks

**DOI:** 10.1371/journal.pone.0307553

**Published:** 2024-07-23

**Authors:** Huayan Pei, Huanmin Wang, Guanghui Yan

**Affiliations:** 1 School of Electronic and Information Engineering, Lanzhou Jiaotong University, Lanzhou, Gansu, China; 2 School of Mechanical Engineering, Lanzhou Jiaotong University, Lanzhou, Gansu, China; Southwest University, CHINA

## Abstract

Despite much progress in exploring the coupled epidemic-awareness dynamics in multiplex networks, little attention has been paid to the joint impacts of behavioral observability and social proof on epidemic spreading. Since both the protective actions taken by direct neighbors and the observability of these actions have essential influence on individuals’ decisions. Thus, we propose a UAPU-SIR model by integrating the effects of these two factors into the decision-making process of taking preventive measures. Specifically, a new state called taken protective actions is introduced into the original unaware-aware-unaware (UAU) model to characterize the action-taken state of individuals after getting epidemic-related information. Using the Microscopic Markov Chain Approach (MMCA), the methods and model are described, and the epidemic threshold is analytically derived. We find that both observability of protecting behaviors and social proof can reduce the epidemic prevalence and raise the epidemic threshold. Moreover, only if observability of protection actions reaches a certain threshold can accelerating information diffusion is able to inhibit disease spreading and result in higher epidemic threshold. We also discover that, reducing the forgetting rate of information is able to decrease epidemic size.

## Introduction

The outburst and transmission of infectious diseases (e.g., smallpox [1], measles [2], Ebola virus [3, 4], and COVID-19 [5–7], etc.) may significantly affect human life and health, and cause economic burden for individuals or countries. When an epidemic breaks out in the population, people’s opinions, discussions and other epidemic-related information spread quickly on various virtual platforms. That is, the outbreak and dissemination of epidemics is commonly followed by information diffusion. Individuals may take certain protective actions such as wearing medical masks, reducing party activities and staying out of crowded places after gaining the infective disease-related information, which in turn may drastically influence epidemic spreading [8–12]. In modelling the impact of human actions on epidemic propagation, the influence of behavioral change is a decisive determinant except the sources of information [13, 14]. Thus, the factors cause behavioral alteration may also affect epidemic dissemination. Consequently, it is extremely crucial to explore the complex interplay patterns between epidemic spreading and behavioral change determinants, which has salient significance for the prevention or control of infectious diseases.

Over the past few decades, there has been growing interest in exploring the coupled epidemic-awareness dynamics within the multiplex network framework [15–27]. Typically, in 2009, Funk et al. investigated the impact of awareness diffusion on epidemic spreading on single-layered network [9]. They revealed that information dissemination is able to decrease the incidence of epidemics, and even inhibit the outburst of epidemics under certain conditions. As pioneers, Granell et al. [15] firstly proposed a UAU-SIS model to explore the dynamical interplay between awareness diffusion and epidemic spreading on multiplex networks, and found that information transmission can help to control the outbreak threshold of epidemic. Later, Granell et al. [16] further studied the mutual effect of information and infective diseases dissemination on multiplex networks by taking the effect of mass media into consideration. Wang et al. [17] constructed an asymmetrically interacting, double-layer network model to analyze the interplay between information diffusion and epidemic spreading. Their results suggested that the outburst of epidemic on a physical-contact network can induce an outbreak of awareness propagation on the communication network, which in turn can efficiently increase the epidemic threshold. Guo et al. [18] uncovered the effect of awareness diffusion on the outburst of epidemic in multiplex networks, and showed that the community information ratio has two-stage impacts on epidemic threshold and results in distinct final epidemic sizes. Besides, Wang et al. [19] explored the propagation dynamics between information diffusion and disease contagion, and uncovered that there exist asymmetrical interactions between the two processes.

In particular, Zheng et al. [20] proposed a UAU-SIR model to investigate the interplay between disease propagation and awareness dissemination in multiplex networks. Moreover, Zhan et al. [21] investigated the coupling dynamics of epidemic diffusion and awareness dissemination, and revealed that epidemic spreading favors information propagation, which in turn suppresses the transmission of infectious disease. It is worth noting that Xia et al. [22] presented a UAU-SIR model to uncover the impacts of mass media on epidemic transmission. Wang et al. [23] and Kabir et al. [24] proposed a UAU-SIR model to uncover the impact of awareness diffusion on epidemic transmission, and the findings demonstrated that the epidemic threshold is closely associated with the propagation of information and the topology of physical-contact network. Kabir and Tanimoto [25] established an SIR-UAU model to discover the role of information dissemination on disease spreading under diverse network structures. Besides, Wang et al. [26, 27] further investigated the impact of two opposing information on epidemic spreading in the absence and presence of mass media. They concluded that these two kinds of information have contrary impacts on epidemic prevention and control. Additionally, Feng et al. [28] uncovered how individuals with different properties in the information diffusion layer influences the transmission of epidemics. These investigations suggest that epidemic spreading is highly related to the relevant information diffusion.

In the real world, generally, the type of information on which people base their decisions is locally available information, but not the globally available one. Since we commonly care more about the decisions made by our friends or acquaintances rather than others in the population. That is to say, in most cases, the choices of our immediate neighbors have decisive impacts on our options [29], and may ultimately result in a behavioral change. This phenomenon is called social proof by psychologists, which is defined as the situation in which an individual needs various inspire from neighbors before adopting a behavior or an opinion [30–32]. Most importantly, social proof requires the behaviors can be observable either directly or via gossip, which is called behavioral observability. In other words, individuals prefer to imitate the actions of their direct neighbors, which is due mainly to the choices of others may provide effective reference for us [29]. As a result, when an epidemic breaks out in the population, whether to or take what protective measures is strongly associated with the observable actions have been taken by nearest neighbors [33].

Motivated by these research, we propose a UAPU-SIR model by incorporating the impacts of behavioral observability and social proof into the decision-making process of taking preventing actions. A new state called taken protective actions (abbreviated as P) is introduced into the classical UAU model to characterize the action-taken state of individuals after gaining disease-related information. The influence of social proof in online shopping has been previously investigated [34, 35], as well as the effect of behavioral observability on prosocial behavior [36, 37]. However, to the best of our knowledge, the impacts of behavioral observability and social proof on epidemic spreading has not been previously reported. In our work, the probability of aware-state individuals to take defensive measures and change into P-state is jointly decided by these two factors.

In the proposed model, a two-layer multiplex network is used to characterize the coupled interaction process between information and epidemic. The upper layer denotes the diffusion of information, and the lower one represents the transmission of epidemic. The Microscopic Markov Chain Approach (MMCA) is utilized to theoretically analyze our model. Here, our focus is to explore the effects of behavioral observability, social proof, as well as information diffusion on epidemic spreading scale, speed and the outburst threshold. Simulation results show that under the proposed model, the observability of immediate neighbors’ protecting actions, the decisions of nearby neighbors (the social proof), as well as the diffusion of information greatly inhibit epidemic contagion, and lead to higher epidemic threshold. It is noteworthy that the longer the disease-related information exists in the population, the more obvious the inhibition effect of our model on epidemic spreading.

This paper is arranged as below. Materials and methods section describes the co-evolutionary spreading model in detail, and analytically derives the epidemic threshold. Simulation results and discussions section presentes and discusses the simulation results. Conclusion section gives a summary of this work.

## Materials and methods

### Model description

**Fig 1:**
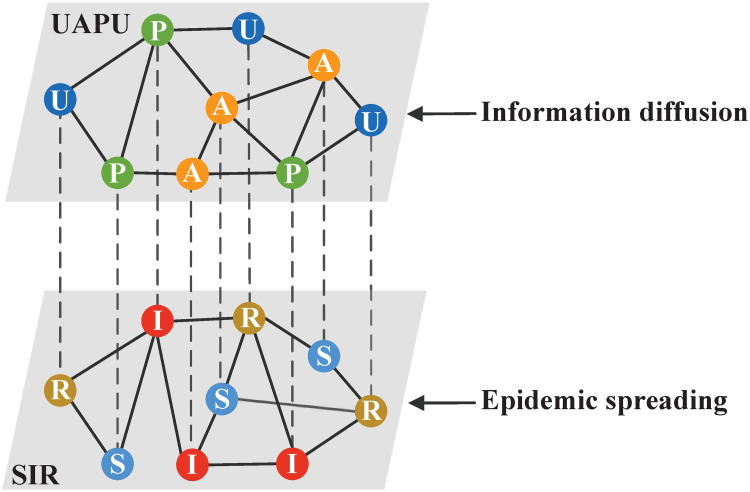
Schematic diagram of the two-layer coupled network employed in the UAPU-SIR model. The procedure of information diffusion is illustrated in the upper layer. The process of epidemic propagation is exhibited in the lower layer. The green nodes in the upper layer denote the individuals who take self-protective actions.

**Fig 2:**
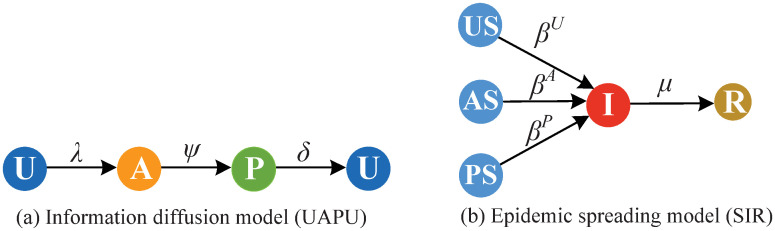
The state transition schematic of information diffusion [panel(a)] and epidemic transmission [panel(b)].

In this work, a two-layer coupled network is used to portray the co-evolution propagation between epidemic and information, as shown in Fig 1. The upper layer is the information diffusion network, which is a virtual network such as Weibo or Facebook. The lower one is the epidemic spreading network which is a physical contact network. In the information transmission layer, the proposed UAPU (unaware-aware-taken protective actions-unaware) model is used. To be specific, a new state called taken protective actions (abbreviated as P) is incorporated into the traditional UAU model to describe the status that an individual has taken prevention measures. Individuals in states U do not have disease-related information [15], hence, they will not take epidemic prevention actions. Each individual in states A has epidemic-related information, but does not take any preventive actions. The disease-related information can be obtained with the probability λ through two sources, one is the individual infected the disease in the lower layer, the other is he has communication with A-state or P-state neighbors in the upper layer. Notably, individuals in states A have a certain possibility *ψ* (*ψ* ∈ [0, 1]) to take prevention measures and change into P-state. For P-state individuals, the forgetting rate of information is *δ*. In the lower layer, the classical SIR (susceptible-infected-susceptible) model is applied to describe the process of epidemic spreading. The state transition schematic of the proposed model is illustrated in Fig 2.

In our model, apart from the initial five states in the UAU-SIR model, three new different statuses are introduced: taken protective action and susceptible (PS); taken protective action and infected (PI); taken protective action and recovered (PR). Noteworthy, an individual will instantly change into AI state once he has infected the epidemic. Therefore, the state UI is spurious.

In the real world, how people respond to the sudden outburst of epidemic not only depend on the locally available information, but also the observability of protective behaviors that have been taken by their immediate neighbors. In other words, the observable actions of those around us plays an essential role in our behavioral change. Inspired by this fact, we integrate behavioral observability and social proof into the coupled awareness-disease model, and present a UAPU-SIR model. More explicitly, the probability of an A-state individual changes into P-state is jointly decided by two factors. One is the observability of nearby neighbors’ protective actions, which is represented by the behavioral observability factor *η*. The other is the decisions of immediate neighbors, which is denoted by the fraction of P-state neighbors. That is, the more neighbors taking protecting measures, the higher the possibility for an individual to transform from A-state to P-state, which well reflects the influence of social proof on individuals’ decisions. Thus, the probability *ψ* that an individual takes protective actions and changes into P-state can be calculated as

$$\begin{array}{c} \psi =log_{2}\left[1+p*\eta +\left(1-p\right)*\frac{\sum_{j}a_{ji}P_{j}^{P}\left(t\right)}{k_{i}}\right]. \end{array}$$ (1)

where parameter *p* ∈ [0, 1] denotes the influence intensity’ proportion of behavioral observability and social proof on *ψ*. Parameter *η* ∈ [0, 1] represents the influence strength of behavioral observability on *ψ*. In our model, larger *η* represents greater observability, which implies that with a higher possibility, one’s protective behavior can be observed or learned by others in the same population. Parameter $P_{j}^{P}\left(t\right)$ denotes the possibility that neighbor *j* of individual *i* is in P-state, and *k*_*i*_ denotes the total number of node *i*’s direct neighbors. Parameter *a*_*ji*_ denotes the elements of the adjacency matrix of the upper layer. In the proposed model, A-state individuals do not take any prevention actions if *ψ* = 0, indicating that information diffusion do not affect epidemic transmission. Conversely, P-state individuals are completely immune to epidemic spreading when *ψ* = 1.

The epidemic infection rate of individuals in U-state, A-state and P-state are represented as *β*^*U*^, *β*^*A*^ and *β*^*P*^ respectively, where *β*^*U*^ = *β*, *β*^*A*^ = *γβ*, and *β*^*P*^ = *log*_2_(2 − *ψ*)*β*. In other words, the infectivity of individuals in AS state will be decreased by a factor *γ* (*γ* ∈ [0, 1]), which is set to be 1 in our model. While, for individuals in PS state, the contagion rate will be reduced by a factor *log*_2_(2 − *ψ*)(*log*_2_(2 − *ψ*) ∈ [0, 1]).

### Theoretical analysis

In this section, the MMCA is utilized to analyze the dynamical interaction between information diffusion and epidemic propagation. We define *A* = (*a*_*ij*_) and *B* = (*b*_*ij*_) as the adjacency matrix of the upper and lower layers. At time step *t*, the possibilities of individual *i* being in each state is denoted as $p_{i}^{US}\left(t\right)$, $p_{i}^{UR}\left(t\right)$, $p_{i}^{AS}\left(t\right)$, $p_{i}^{AI}\left(t\right)$, $p_{i}^{AR}\left(t\right)$, $p_{i}^{PS}\left(t\right)$, $p_{i}^{PI}\left(t\right)$, and $p_{i}^{PR}\left(t\right)$, respectively. Noteworthy, the dynamical correlations are assumed to be absent [38]. In the upper layer, the probability that an individual *i* in status US(UR) is not aware of epidemic-related information is *r*_*i*_(*t*). For the epidemic dissemination, the probability that an individual *i* in states U, A and P will not be infected at time step *t* is defined as $q_{i}^{U}\left(t\right)$, $q_{i}^{A}\left(t\right)$ and $q_{i}^{P}\left(t\right)$, respectively. As a consequence, *r*_*i*_(*t*), $q_{i}^{U}\left(t\right)$, $q_{i}^{A}\left(t\right)$ and $q_{i}^{P}\left(t\right)$ can be calculated as

$$\begin{array}{cc} & r_{i}\left(t\right)=\prod_{j}\left[1-a_{ji}p_{j}^{AP}\left(t\right)\lambda \right], \end{array}$$ (2a)

$$\begin{array}{cc} & q_{i}^{U}\left(t\right)=\prod_{j}\{1-b_{ji}p_{j}^{I}\left(t\right)\beta^{U}\}, \end{array}$$ (2b)

$$\begin{array}{cc} & q_{i}^{A}\left(t\right)=\prod_{j}\{1-b_{ji}p_{j}^{I}\left(t\right)\beta^{A}\}, \end{array}$$ (2c)

$$\begin{array}{cc} & q_{i}^{P}\left(t\right)=\prod_{j}\{1-b_{ji}p_{j}^{I}\left(t\right)\beta^{P}\}, \end{array}$$ (2d)

where $p_{j}^{AP}\left(t\right)=p_{j}^{AS}\left(t\right)+p_{j}^{AI}\left(t\right)+p_{j}^{AR}\left(t\right)+p_{j}^{PS}\left(t\right)+p_{j}^{PI}\left(t\right)+p_{j}^{PR}\left(t\right)$, and $p_{j}^{I}\left(t\right)=p_{j}^{AI}\left(t\right)+p_{j}^{PI}\left(t\right)$.

**Fig 3:**
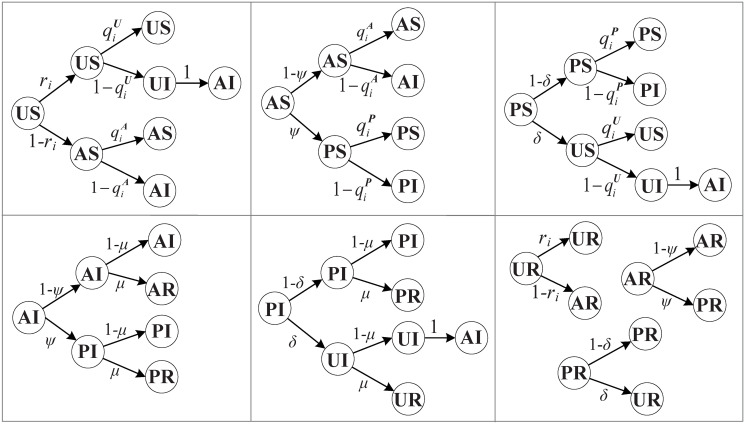
Transition probability trees of US, AS, PS, AI, PI, UR, AR, PR in the UAPU-SIR model.

We establish the Markov state transition trees (as shown in Fig 3) to describe the possible transformations of each status, combining with Eqs (2a)–(2d), the MMCA equations for every node *i* at time step *t* can be attained as below

$$\begin{array}{c} p_{i}^{US}\left(t+1\right)=p_{i}^{US}\left(t\right)r_{i}\left(t\right)q_{i}^{U}\left(t\right)+p_{i}^{PS}\left(t\right)\delta q_{i}^{U}\left(t\right), \end{array}$$ (3a)

$$\begin{array}{c} p_{i}^{AS}\left(t+1\right)=p_{i}^{US}\left(t\right)\left[1-r_{i}\left(t\right)\right]q_{i}^{A}\left(t\right)+p_{i}^{AS}\left(t\right)\left(1-\psi \right)q_{i}^{A}\left(t\right), \end{array}$$ (3b)

$$\begin{array}{c} p_{i}^{PS}\left(t+1\right)=p_{i}^{AS}\left(t\right)\psi q_{i}^{P}\left(t\right)+p_{i}^{PS}\left(t\right)\left(1-\delta \right)q_{i}^{P}\left(t\right), \end{array}$$ (3c)

$$\begin{array}{cc} p_{i}^{AI}\left(t+1\right) & =p_{i}^{US}\left(t\right)r_{i}\left(t\right)\left[1-q_{i}^{U}\left(t\right)\right]+p_{i}^{US}\left(t\right)\left[1-r_{i}\left(t\right)\right]\left[1-q_{i}^{A}\left(t\right)\right]\\ & +p_{i}^{AS}\left(t\right)\left(1-\psi \right)\left[1-q_{i}^{A}\left(t\right)\right]+p_{i}^{PS}\left(t\right)\delta \left[1-q_{i}^{U}\left(t\right)\right]\\ & +p_{i}^{AI}\left(t\right)\left(1-\psi \right)\left(1-\mu \right)+p_{i}^{PI}\left(t\right)\delta \left(1-\mu \right), \end{array}$$ (3d)

$$\begin{array}{cc} p_{i}^{PI}\left(t+1\right) & =p_{i}^{AS}\left(t\right)\psi \left[1-q_{i}^{P}\left(t\right)\right]+p_{i}^{PS}\left(t\right)\left(1-\delta \right)\left[1-q_{i}^{P}\left(t\right)\right]\\ & +p_{i}^{AI}\left(t\right)\psi \left(1-\mu \right)+p_{i}^{PI}\left(t\right)\left(1-\delta \right)\left(1-\mu \right), \end{array}$$ (3e)

$$\begin{array}{c} p_{i}^{UR}\left(t+1\right)=p_{i}^{UR}\left(t\right)r_{i}\left(t\right)+p_{i}^{PR}\left(t\right)\delta +p_{i}^{PI}\left(t\right)\delta \mu , \end{array}$$ (3f)

$$\begin{array}{c} p_{i}^{AR}\left(t+1\right)=p_{i}^{AI}\left(t\right)\left(1-\psi \right)\mu +p_{i}^{UR}\left(t\right)\left[1-r_{i}\left(t\right)\right]+p_{i}^{AR}\left(t\right)\left(1-\psi \right), \end{array}$$ (3g)

$$\begin{array}{c} p_{i}^{PR}\left(t+1\right)=p_{i}^{AI}\left(t\right)\psi \mu +p_{i}^{AR}\left(t\right)\psi +p_{i}^{PR}\left(1-\delta \right)+p_{i}^{PI}\left(t\right)\left(1-\delta \right)\mu , \end{array}$$ (3h)

where $p_{i}^{US}\left(t\right)+p_{i}^{AS}\left(t\right)+p_{i}^{PS}\left(t\right)+p_{i}^{AI}\left(t\right)+p_{i}^{PI}\left(t\right)+p_{i}^{UR}\left(t\right)+p_{i}^{AR}\left(t\right)+p_{i}^{PR}\left(t\right)=1$.

When epidemic spreading reaches the stationary state, $p_{i}^{US}\left(t+1\right)=p_{i}^{US}\left(t\right)=p_{i}^{US}$ is satisfied for each node *i* and correspondingly for the other seven states. Near the critical point of the epidemic threshold, the infection probability of every node approaches 0. Hence, it can be set that $p_{i}^{I}=p_{i}^{AI}+p_{i}^{PI}=\epsilon_{i}\ll 1$. Consequently, Eqs (2b)–(2d) can be approximately changed into

$$\begin{array}{cc} & q_{i}^{U}\approx 1-\beta^{U}\sum_{j}b_{ji}\epsilon_{j}=1-\xi_{i}, \end{array}$$ (4a)

$$\begin{array}{cc} & q_{i}^{A}\approx 1-\gamma \beta^{U}\sum_{j}b_{ji}\epsilon_{j}=1-\gamma \xi_{i}, \end{array}$$ (4b)

$$\begin{array}{cc} & q_{i}^{P}\approx 1-log_{2}\left(2-\psi \right)\beta^{U}\sum_{j}b_{ji}\epsilon_{j}=1-log_{2}\left(2-\psi \right)\xi_{i}, \end{array}$$ (4c)

where

$$\begin{array}{c} \xi_{i}=\beta^{U}\sum_{j}b_{ji}\epsilon_{j}. \end{array}$$ (5)

Inserting Eqs (4a)–(4c) into Eqs (3a)–(3e), we obtain

$$\begin{array}{c} p_{i}^{US}\left(t+1\right)=p_{i}^{US}\left(t\right)r_{i}\left(t\right)\left(1-\xi_{i}\right)+p_{i}^{PS}\left(t\right)\delta \left(1-\xi_{i}\right), \end{array}$$ (6a)

$$\begin{array}{c} p_{i}^{AS}\left(t+1\right)=p_{i}^{US}\left(t\right)\left[1-r_{i}\left(t\right)\right]\left(1-\gamma \xi_{i}\right)+p_{i}^{AS}\left(t\right)\left(1-\psi \right)\left(1-\gamma \xi_{i}\right), \end{array}$$ (6b)

$$\begin{array}{cc} p_{i}^{PS}\left(t+1\right) & =p_{i}^{AS}\left(t\right)\psi \left[1-log_{2}\left(2-\psi \right)\xi_{i}\right]\\ & +p_{i}^{PS}\left(t\right)\left(1-\delta \right)\left[1-log_{2}\left(2-\psi \right)\xi_{i}\right], \end{array}$$ (6c)

$$\begin{array}{cc} p_{i}^{AI}\left(t+1\right) & =p_{i}^{US}\left(t\right)r_{i}\left(t\right)\xi_{i}+p_{i}^{US}\left(t\right)\left[1-r_{i}\left(t\right)\right]\gamma \xi_{i}\\ & +p_{i}^{AS}\left(t\right)\left(1-\psi \right)\gamma \xi_{i}+p_{i}^{PS}\left(t\right)\delta \xi_{i}\\ & +p_{i}^{AI}\left(t\right)\left(1-\psi \right)\left(1-\mu \right)+p_{i}^{PI}\left(t\right)\delta \left(1-\mu \right), \end{array}$$ (6d)

$$\begin{array}{cc} p_{i}^{PI}\left(t+1\right) & =p_{i}^{AS}\left(t\right)\psi log_{2}\left(2-\psi \right)\xi_{i}+p_{i}^{PS}\left(t\right)\left(1-\delta \right)log_{2}\left(2-\psi \right)\xi_{i}\\ & +p_{i}^{AI}\left(t\right)\psi \left(1-\mu \right)+p_{i}^{PI}\left(t\right)\left(1-\delta \right)\left(1-\mu \right). \end{array}$$ (6e)

Meanwhile, when the system is at equilibrium, by adding Eqs (6d) and (6e), we can get

$$\begin{array}{cc} p_{i}^{AI}+p_{i}^{PI} & =\left(1-\mu \right)\left(p_{i}^{AI}+p_{i}^{PI}\right)+\xi_{i}\left(p_{i}^{US}r_{i}+p_{i}^{PS}\delta \right)\\ & +\gamma \xi_{i}\left[p_{i}^{US}\left(1-r_{i}\right)+p_{i}^{AS}\left(1-\psi \right)\right]\\ & +log_{2}\left(2-\psi \right)\xi_{i}\left[p_{i}^{AS}\psi +p_{i}^{PS}\left(1-\delta \right)\right]. \end{array}$$ (7)

When *β* → *β*_*c*_, we can get $p_{i}^{AI}\to 0,p_{i}^{PI}\to 0,p_{i}^{UR}\to 0,p_{i}^{AR}\to 0$, and $p_{i}^{PR}\to 0$. Thus, it can be acquired that $p_{i}^{U}=p_{i}^{US}+p_{i}^{UR}\approx p_{i}^{US}$, $p_{i}^{A}=p_{i}^{AS}+p_{i}^{AI}+p_{i}^{AR}\approx p_{i}^{AS}$, and $p_{i}^{P}=p_{i}^{PS}+p_{i}^{PI}+p_{i}^{PR}\approx p_{i}^{PS}$. Therefore, Eq (7) can be rewritten as

$$\begin{array}{cc} \epsilon_{i} & =\left(1-\mu \right)\epsilon_{i}+\xi_{i}\left(p_{i}^{U}r_{i}+p_{i}^{P}\delta \right)\\ & +\gamma \xi_{i}\left[p_{i}^{U}\left(1-r_{i}\right)+p_{i}^{A}\left(1-\psi \right)\right]\\ & +log_{2}\left(2-\psi \right)\xi_{i}\left[p_{i}^{A}\psi +p_{i}^{P}\left(1-\delta \right)\right]. \end{array}$$ (8)

Further, removing ◯(*ϵ*_*i*_) terms of Eqs (3a)–(3c), it can be obtained that

$$\begin{array}{cc} & p_{i}^{U}=p_{i}^{U}r_{i}+p_{i}^{P}\delta , \end{array}$$ (9a)

$$\begin{array}{cc} & p_{i}^{A}=p_{i}^{U}\left(1-r_{i}\right)+p_{i}^{A}\left(1-\psi \right), \end{array}$$ (9b)

$$\begin{array}{cc} & p_{i}^{P}=p_{i}^{A}\psi +p_{i}^{P}\left(1-\delta \right). \end{array}$$ (9c)

Incorporating Eqs (9a)–(9c) into Eq (8), we can get

$$\begin{array}{c} \epsilon_{i}=\left(1-\mu \right)\epsilon_{i}+\xi_{i}p_{i}^{U}+\gamma \xi_{i}p_{i}^{A}+log_{2}\left(2-\psi \right)\xi_{i}p_{i}^{P}. \end{array}$$ (10)

Additionally, taking Eq (5) into consideration, Eq (10) can be further rewritten as

$$\begin{array}{c} \sum_{j}\{\left[p_{i}^{U}+\gamma p_{i}^{A}+log_{2}\left(2-\psi \right)p_{i}^{P}\right]b_{ji}-\frac{\mu }{\beta^{U}}\theta_{ji}\}\epsilon_{j}=0, \end{array}$$ (11)

where *θ*_*ij*_ represents the elements of identity matrix. Notably, the solution of Eq (11) reduces to resolve the eigenvalues of matrix *H* whose elements are $h_{ji}=\left[p_{i}^{U}+\gamma p_{i}^{A}+log_{2}\left(2-\psi \right)p_{i}^{P}\right]b_{ji}$. We define the maximum eigenvalue of matrix *H* as Λ_*max*_(*H*), then the epidemic threshold is

$$\begin{array}{c} \beta_{c}^{U}=\frac{\mu }{\Lambda_{max}\left(H\right)}. \end{array}$$ (12)

According to Eqs (11) and (12), in our model, the outbreak of epidemic is tightly related to the diffusion of disease-related information, behavioral observability, social proof, and network structure of the epidemic transmission layer.

## Simulation results and discussions

**Fig 4:**
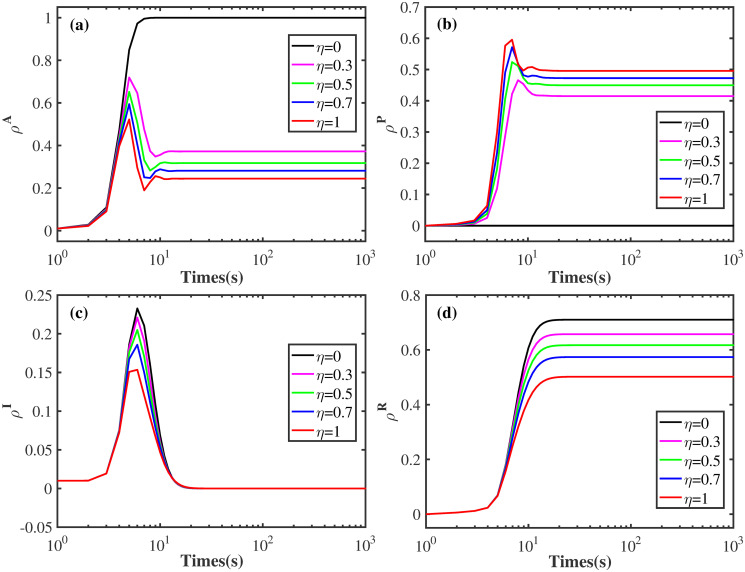
Effect of behavioral observability factor *η* on epidemic size and disease spreading speed from a microscopic point of view. (a) The fraction of A-state individuals *ρ*^*A*^. (b) The proportion of protective action taken nodes *ρ*^*P*^. (c) The percentage of infected individuals *ρ*^*I*^. (d) The fraction of recovered nodes *ρ*^*R*^. Here, λ = 0.3, *δ* = 0.4, *β* = 0.2, *μ* = 0.6, and *p* = 0.5. Transmission speed, epidemic size are both inhibited for rising *η*, while *ρ*^*A*^, *ρ*^*I*^, *ρ*^*R*^ and the corresponding critical value are greatly decreased.

**Fig 5:**
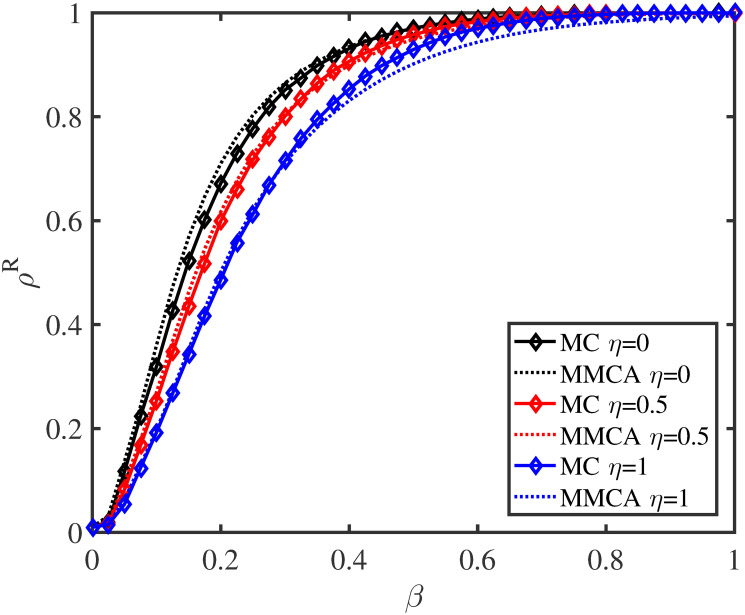
Influence of *η* on disease size and epidemic transmission speed. Here, λ = 0.3, *δ* = 0.4, *μ* = 0.6, and *p* = 0.5.

**Fig 6:**
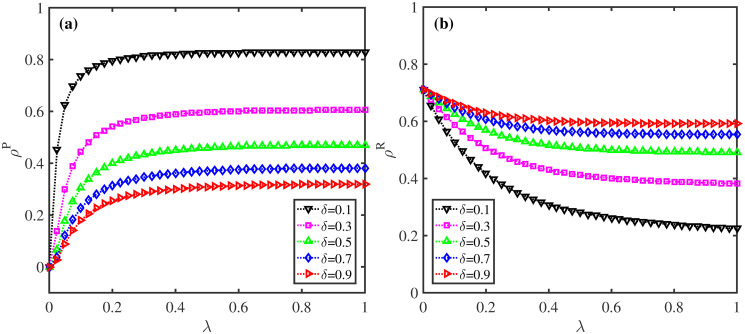
Impact of information diffusion rate λ and information forgetting rate *δ* on *ρ*^*P*^ and *ρ*^*R*^. (a) The fraction of preventive action taken nodes *ρ*^*P*^; (b) The density of recovered nodes *ρ*^*R*^. Here, *η* = 1, *β* = 0.2, *μ* = 0.6, and *p* = 0.5.

**Fig 7:**
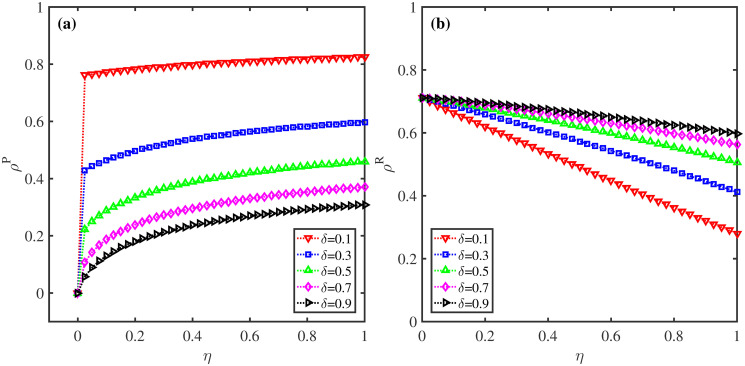
Effect of behavioral observability factor *η* and information forgetting rate *δ* on *ρ*^*P*^ and *ρ*^*R*^. (a) The proportion of protective action taken individuals *ρ*^*P*^; (b) The fraction of recovered nodes *ρ*^*R*^. Here, λ = 0.5, *β* = 0.2, *μ* = 0.6, and *p* = 0.5.

**Fig 8:**
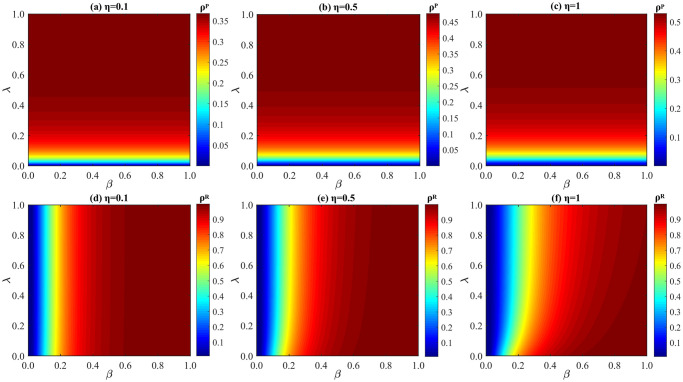
Impact of λ and *β* on *ρ*^*P*^ and *ρ*^*R*^ under distinct values of *η*. (a) *ρ*^*P*^ when *η* = 0.1; (b) *ρ*^*P*^ when *η* = 0.5; (c) *ρ*^*P*^ when *η* = 1; (d) *ρ*^*R*^ when *η* = 0.1; (e) *ρ*^*R*^ when *η* = 0.5; (f) *ρ*^*R*^ when *η* = 1. The parameters: *δ* = 0.4, *μ* = 0.6, and *p* = 0.5.

**Fig 9:**
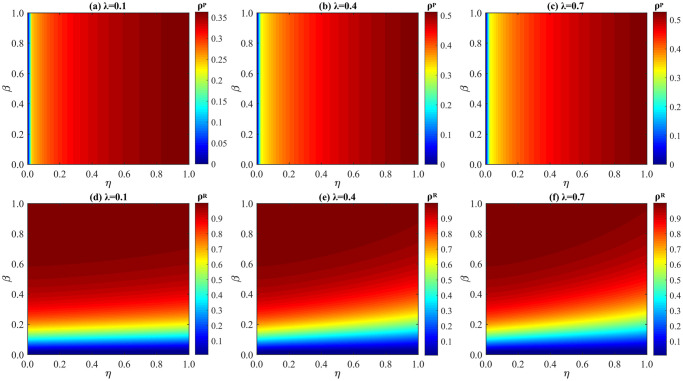
Influence of *η* and *β* on *ρ*^*P*^ and *ρ*^*R*^ under different values of λ. (a) *ρ*^*P*^ when λ = 0.1; (b) *ρ*^*P*^ when λ = 0.4; (c) *ρ*^*P*^ when λ = 0.7; (d) *ρ*^*R*^ when λ = 0.1; (e) *ρ*^*R*^ when λ = 0.4; (f) *ρ*^*R*^ when λ = 0.7. Here, *δ* = 0.4, *μ* = 0.6, and *p* = 0.5.

**Fig 10:**
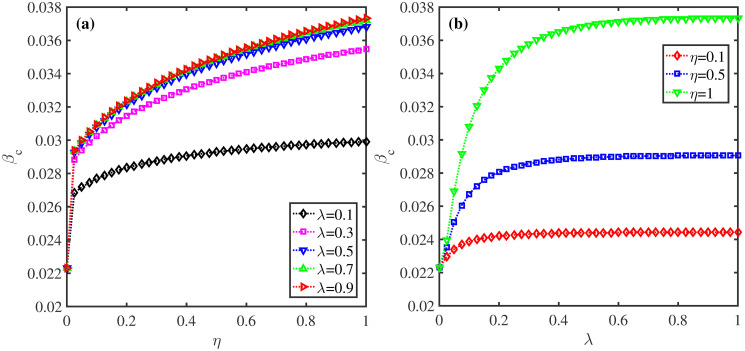
Effect of *η* and λ on epidemic outbreak threshold. (a) The epidemic threshold *β*_*c*_ as a function of *η* under different values of λ. (b) The epidemic threshold *β*_*c*_ as a function of λ for distinct values of *η*. The parameters: *δ* = 0.1, *σ* = 0.2, and *μ* = 0.6.

In this work, two Barabsi-Albert (BA) networks are constructed on the upper and lower layers, the reconnected number m of the corresponding networks are 4 and 3, respectively. The size of each network is *N* = 3 × 10^3^. Here, MMCA and Monte Carlo (MC) are applied to carry out the simulations. For the MMCA, *ρ*^*R*^, *ρ*^*A*^, *ρ*^*I*^, *ρ*^*P*^ are respectively calculated as $\rho^{R}=\frac{1}{N}\sum_{i=1}^{N}\left(p_{i}^{UR}+p_{i}^{AR}+p_{i}^{PR}\right)$, $\rho^{A}=\frac{1}{N}\sum_{i=1}^{N}\left(p_{i}^{AS}+p_{i}^{AI}+p_{i}^{AR}\right)$, $\rho^{I}=\frac{1}{N}\sum_{i=1}^{N}\left(p_{i}^{AI}+p_{i}^{PI}\right)$, $\rho^{P}=\frac{1}{N}\sum_{i=1}^{N}\left(p_{i}^{PS}+p_{i}^{PI}+p_{i}^{PR}\right)$. Nevertheless, in MC simulation, *ρ*^*R*^ is computed as $\rho^{R}=\frac{N_{R}}{N}$, where *N*^*R*^ represents the total number of R-state individuals in the population. Let *ρ*^*AI*^ and *ρ*^*US*^ represent the percentage of nodes in status AI and US, respectively. Originally, it is set *ρ*^*AI*^ = 0.01 and *ρ*^*US*^ = 0.99. Additionally, each data spot is acquired by averaging over 50 independent runs. In Fig 5, the results are obtained by MMCA and MC, and in Figs 4, 6 to 10, all the results are gained by MMCA.

Fig 4 explores the influence of behavioral observability and social proof on disease size and spreading speed from a microscopic point of view. It can be found that the values of *ρ*^*P*^ shows an obvious rise when *η* is increased from 0 to 0.3, and higher *ρ*^*P*^ can be observed with the further increase of *η*. This result suggests that when A-state individuals can observe the protective actions taken by direct neighbors, they are more inclined to take the same preventive measures. Conversely, the values of *ρ*^*A*^, *ρ*^*I*^ and *ρ*^*R*^ all display a salient drop with rising *η*, indicating that the protecting actions resulted from behavioral observability and social proof can suppress the epidemic size and transmission speed.

Fig 5 uncovers the changes of *ρ*^*R*^ with epidemic infection rate *β* when behavioral observability factor *η* takes different values, and compares the results of MMCA and MC. We find that compared to the value of *ρ*^*R*^ when *η* = 0, an obvious decrease can be seen for *η* = 1, which validates that increasing the observability of nearby neighbors’ preventive actions can inhibit epidemic propagation. Besides, the findings demonstrate that the results of MMCA and MC have a good consistency.

Fig 6 examines the changes of *ρ*^*P*^ and *ρ*^*R*^ with information diffusion rate λ when information forgetting rate *δ* takes distinct values. It can be detected that, for λ ≤ 0.3, greater *ρ*^*P*^ corresponds to larger values of λ, which indicates that the faster the information spreads, the more individuals taking protecting measures. However, increment of *δ* suppresses the influence of information dissemination on the emergence of protective behaviors. Moreover, for fixed *δ*, greater λ leads to an obvious decrease of *ρ*^*R*^, which implies that in our model, the spreading of information greatly inhibits epidemic size. Whereas, for increasing *δ*, the inhibition effect of information diffusion on epidemic transmission is reduced.

Fig 7 unveils the changes of *ρ*^*P*^ and *ρ*^*R*^ with behavioral observability factor *η* when information disremembering rate *δ* takes different values. Notably, for fixed *δ*, when protective behaviors are observable, the values of *ρ*^*P*^ exhibits a dramatic increase with rising *η*. In contrast, the values of *ρ*^*R*^ shows an obvious decrease, which further confirms the essential role of behavioral observability and social proof in suppressing epidemic spreading. Furthermore, the greater *δ* is, the smaller *ρ*^*P*^ and higher *ρ*^*R*^ can be observed, which is due mainly to the fact that the faster epidemic-related information disappears, the fewer individuals will obtain the information in the whole population. Individuals tend to take protective measures only if they get the disease-related information and observe others around have taken corresponding protecting actions. These findings suggest that decreasing disremembering rate of information can lead to smaller epidemic size.

We discuss the influence of behavioral observability factor *η* on *ρ*^*P*^ and *ρ*^*R*^ in Fig 8. Clearly, when *η* is risen from 0.1 [panel (d)] to 1 [panel (f)], the values of *ρ*^*R*^ reflects an obvious drop, which demonstrates that epidemic spreading is significantly inhibited when *η* is increased. It is mainly because of more individuals will imitate the protective measures when the observability of preventive actions is risen, which also results in the increase of P-state individuals (as shown in panel(a) to panel(c)). Besides, it is worth noting that only when the observability of neighbors’ protective actions reaches a certain level, epidemic spreading can be inhibited by accelerating information diffusion. Whereas, if the behavioral observability factor *η* is small, such as *η* = 0.1, accelerating information dissemination does not have significant impacts on suppressing disease transmission.

We scrutinize the impact of information diffusion rate λ on *ρ*^*P*^ and *ρ*^*R*^ in Fig 9. Notably, with λ fixed, the greater *η* is, the higher the probability of individuals imitating the observable protective actions under the influence of social proof. Most importantly, the density of R-state individuals is greatly decreased when λ is risen from 0.1 [panel (d)] to 0.7 [panel (f)], which verifies that when information spreads faster, driven by behavioral observability, more individuals are inclined to imitate immediate neighbors and take protecting actions, which results in smaller disease spreading size. However, it also can be found that when λ ≥ 0.4, with the increase of *η*, *ρ*^*P*^ does not show an obvious change, which is mainly because of the influence of social proof. Since the percentage of P-state individuals is jointly decided by behavioral observability and social proof (as shown in Eq 1). To sum up, when λ ≤ 0.4, under the joint effects of behavioral observability and social proof, the faster the information spreads, the more obvious the suppression effects of our model on epidemic size.

Finally, we investigate the changes of *β*_*c*_ with *η* and λ in Fig 10. We find that *β*_*c*_ significantly increases with rising *η* for fixed λ, indicating that increasing observability of neighbors’ protective actions promotes epidemic threshold. Nevertheless, when λ ≥ 0.5, further increment of information dissemination does not result in higher epidemic threshold. Since in such a situation, most individuals in the population have obtained the disease-related information. Meanwhile, in Fig 10(b), it can be observed that *β*_*c*_ exhibits an increase with rising λ for fixed *η*, and the larger *η* is, the more obvious the change of *β*_*c*_ with λ, which further validates that behavioral observability can raise the epidemic threshold.

## Conclusion

During the awareness-epidemic coupled spreading process, both the protective actions taken by direct neighbors and the observability of these actions have crucial influence on our decisions. To uncover the impacts of behavioral observability and social proof on epidemic propagation, we propose a UAPU-SIR model by incorporating the effects of these two factors into the decision-making process of taking protection measures. More explicitly, a new state called taken protective actions is introduced into the classical UAU model to describe the action-taken status of individuals after getting disease-related information. The MMCA is applied to theoretically analyze the presented model and derive the epidemic threshold. To the best of our knowledge, the impacts of behavioral observability and social proof on epidemic spreading has not been previously reported. By theoretical analysis and simulation, we show that the observability of preventive actions and social proof suppress dissemination speed and outbreak size of infective disease, and cause higher epidemic threshold. Furthermore, under the proposed model, only if the observability of protective behavior reaches a certain level, can accelerating information diffusion inhibit epidemic transmission and raise epidemic threshold. We also found that, decreasing the disremembering rate of information can reduce epidemic size.

Our results may offer some insight into predicting and controlling the actual spreading process of epidemic. Nevertheless, in the present model, the behavioral spreading resulting from information diffusion has not been taken into consideration, which also plays an important role in epidemic spreading. In the future work, a more sophisticated UAPU-SEIR model taking into account behavioral propagation will be the focus of our investigation.
